# A pilot study to investigate if the mesenteric circumferential location of colon cancer affects survival when compared to the anti-mesenteric side

**DOI:** 10.1038/s41598-021-88320-6

**Published:** 2021-05-17

**Authors:** E. Horwell, F. Sagias

**Affiliations:** 1 Department of Colorectal Surgery, Oxford University Hospital Trust, Oxford, UK; 2 Department of Colorectal Surgery, Portsmouth Hospitals NHS Trust, Portsmouth, UK; 3 Colorectal Department, Churchill Hospital, Old Rd, Oxford, OX3 7LE UK

**Keywords:** Cancer microenvironment, Gastrointestinal cancer, Gastrointestinal diseases, Surgical oncology

## Abstract

Colorectal cancer is a leading cause of death in the western world. The main datum that is employed to guide treatment and prognosis are related to the pathological stage and the genetics of the cancer. Recent electron-microscopic study of the colonic border has suggested a difference between the micro-anatomy of the mesenteric border^11^, compared to the anti-mesenteric. With colorectal cancer increasing in incidence, the more information that we can employ to guide and tailor patient centred management, the better. A pilot study to test the hypothesis that the circumferential location on the colonic wall, mesenteric or anti-mesenteric, has an impact on the mortality rate associated with right-sided colon cancer. All patients undergoing a right hemicolectomy for non-metastatic adenocarcinoma between 2010 and 2013 were included (155 patients in total). T and N stage were recorded. There was no statistical difference between the groups for age or sex. Survival rates were then calculated according to the location of the cancer and analysed using Kaplan–Meir survival calculations. 100 patients were included in the final analysis. 90 patients had cancer on the antimesenteric border. The T and N stage were not statistically different between the two groups. The mean all-cause survival was 44 months for the mesenteric group and 77 for the antimesenteric (*P* = 0.002). Disease free survival was 41 versus 60 months accordingly (*P* = 0.021). Mesenteric cancer appears to have a shorter survival time, and may be a good candidate for future prognostication and treatment algorithms. Interesting this survival difference is observed even with a lower average T stage in the mesenteric group. The histological recording of the circumferential location is a zero cost and easy metric to record.

## Introduction

Metastases, rather than the primary tumour is responsible for the majority of cancer related deaths^1^. From Paget’s 1889 ‘seed and soil’ hypothesis^2^, our understanding of the pathophysiology of how cancer metastasises is far from complete, but has increased over recent years^3^. For a cancer to metastasise, it requires a series of key dynamic and interconnected steps, simplified in the paradigm of: genetic mutation, cell proliferation, angiogenesis, intravasation, extravasation and finally proliferation at the end organ parenchyma^4–7^. Animal models suggest that for one gram of tumour, 4.6 × 10^6^ tumour cells are shed into the circulation every 24 h. With less than 0.01% of these cells forming secondary tumour growths, most of these cells are rapidly destroyed by the immune system, before they can adhere to distant capillary endothelial layers^2,8^.

One of these vital metastatic steps is stromal invasion. Once this occurs, thin-walled venules or lymphatic channels offer little resistance to penetration by tumour cells, and is regarded as the most common route for tumour cell entry into the systemic circulation^9,10^. A recent study using electronmicroscopy^11^ to analyse the microanatomy of the colon has shown that it is at the mesenteric border that lymphatic networks become endotheliased, more concentrated and start to form the contractile collecting system that leads into the systemic circulation.

With right sided colorectal cancer becoming more prevalent in society, and evidence suggesting it has a higher mortality rate compared to left sided colorectal cancer^12–15^, any further insight into identifying independent prognostication factors to tailor surgical and chemotherapy regimens to the patient is going to be key in reducing mortality.

**Figure 1:**
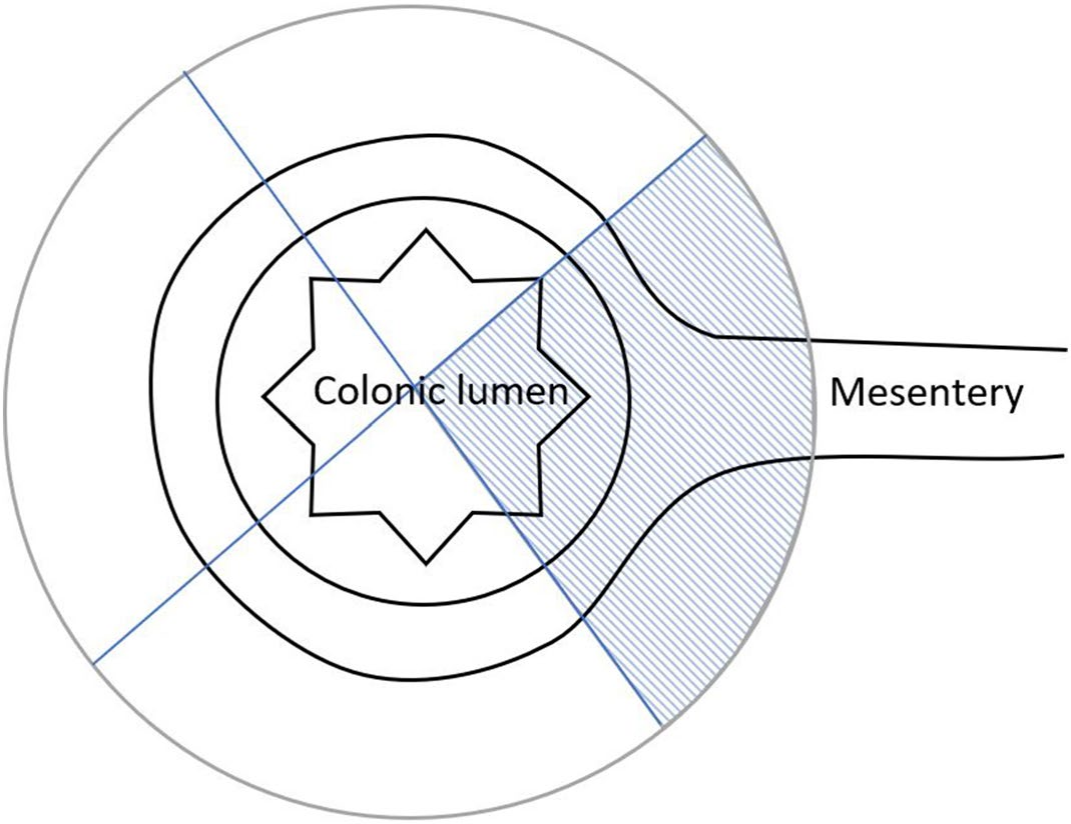
Any tumour in the marked area was considered mesenteric anything outside was considered anti-mesenteric.

Our hypothesis is that if colonic adenocarcinoma develops on the mesenteric border, by virtue of its geography, it is more likely to enter these more ‘developed’ collecting lymphatics and therefore has a higher likelihood of metastasis. This would result in higher mortality rates, when compared to the antimesenteric border (see Fig. 1 for illustration).

For clarification, right sided colon cancer was chosen to be exclusively analysed, not because we think its micro-anatomy or metastatic spread is necessarily different to that on the left, but to avoid any potential confounding factors in the data. We were unable to separate rectal cancer from true left sided colon cancer from the data set for this pilot study. If we were to have included left sided colon cancer in the study, the rectum and its lack of mesenteric border would have added extraneous data, and hence made interpretation difficult.

## Method

**Figure 2:**
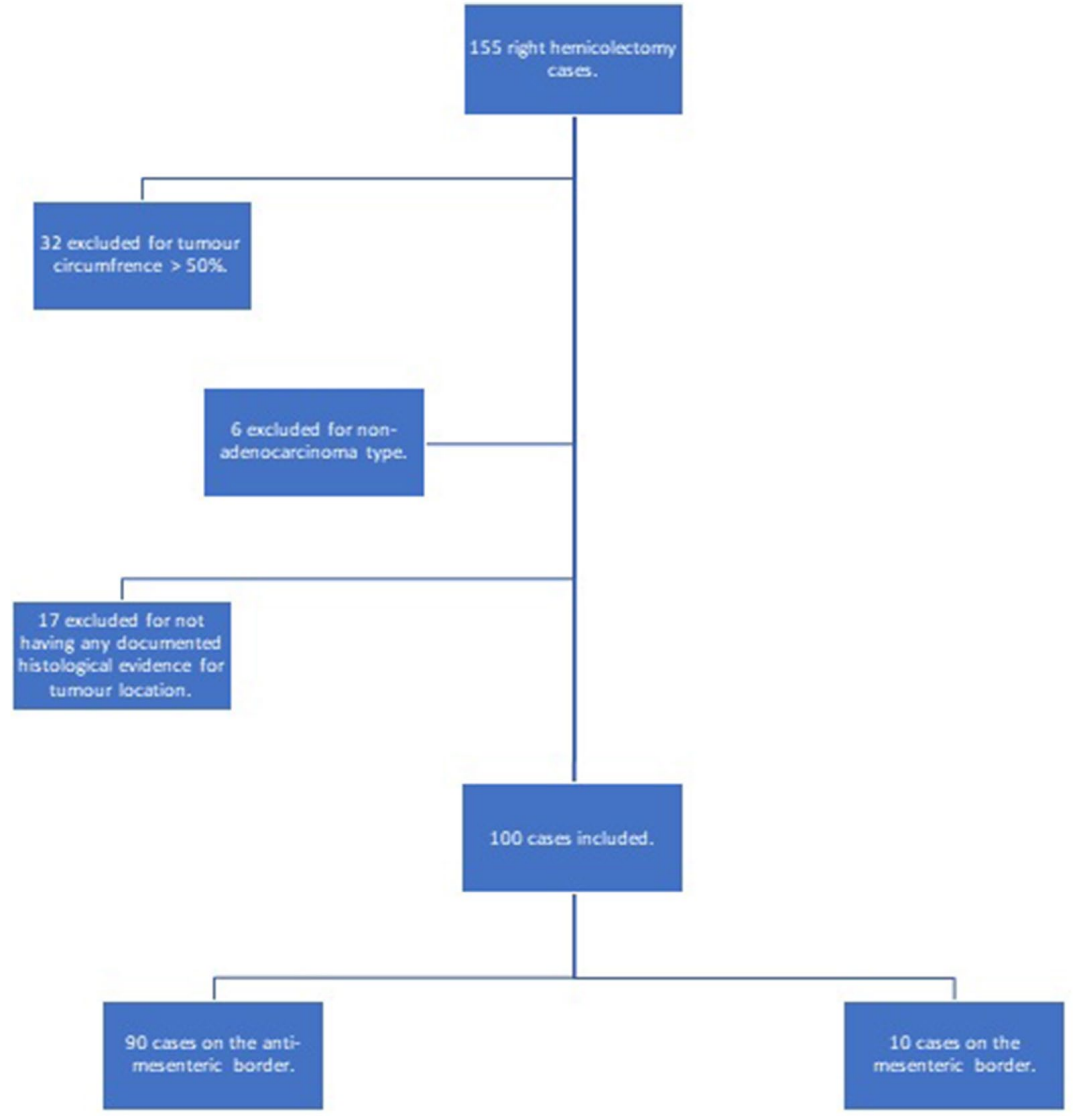
Study inclusion and exclusion criteria flowsheet.

Using a prospectively maintained database of all elective right hemicolectomies performed for malignancy in a large volume district general hospital (Queen Alexandra Hospital, Portsmouth) between January 2010 to June 2013, one hundred and fifty-five patients were identified. The histological reports of these patients were reviewed, and reports categorised into mesenteric, anti-mesenteric or unspecified. Local guidelines on data handling and analysis were followed. The inclusion criteria was for any patient over the age of 18, having a right hemicolectomy for a non-special type (i.e. adenocarcinoma). The exclusion criteria were cases without clear histological reporting on the anatomical border (i.e. mesenteric or otherwise), a total circumference greater than fifty percent or with known pre-operative metastasis (fifty-five cases were excluded by these criteria, see Fig. 2). Each case had its stage, all cause survival and disease-free survival recorded. Using SPSS (IBM V25), Kaplan–Meir survival analysis was performed.

### Human and animal rights

There was no experimentation on humans/human tissue.

### Informed consent

The data used in this study/audit was approved by the Portsmouth Colorectal Audit Department and the local guide lines were followed for data analysis. Informed consent was obtained from all subjects at the time of data collection. No subjects were aged under 18.

## Results

Of the one hundred cases included in this study, ninety were classified as being on the anti-mesenteric border. There were sixty-four females. The average age was 73 (37–91). During the five-year study period there were 32 deaths. There was no statistical difference between T or N stage in the mesenteric versus antimesenteric groups (breakdown of T & N stage can be found in Table 1).

**Table 1:** Patient demographics and stage breakdown.

Demographic	Mesenteric group	Anti-mesenteric group
Age average/(range)	75 (44–84)	73 (37–91)
Female sex	6 (60%)	58 (64%)
**T stage**		
1	1 (10%)	7 (7.8%)
2	3 (30%)	13 (14.4%)
3	6 (60%)	56 (62.2%)
4	0 (0%)	14 (15.5%)
**N stage**		
0	7 (70%)	52 (57.8%)
1	1 (10%)	21 (23.3%)
2	2 (20%)	17 (18.9%)

**Figure 3:**
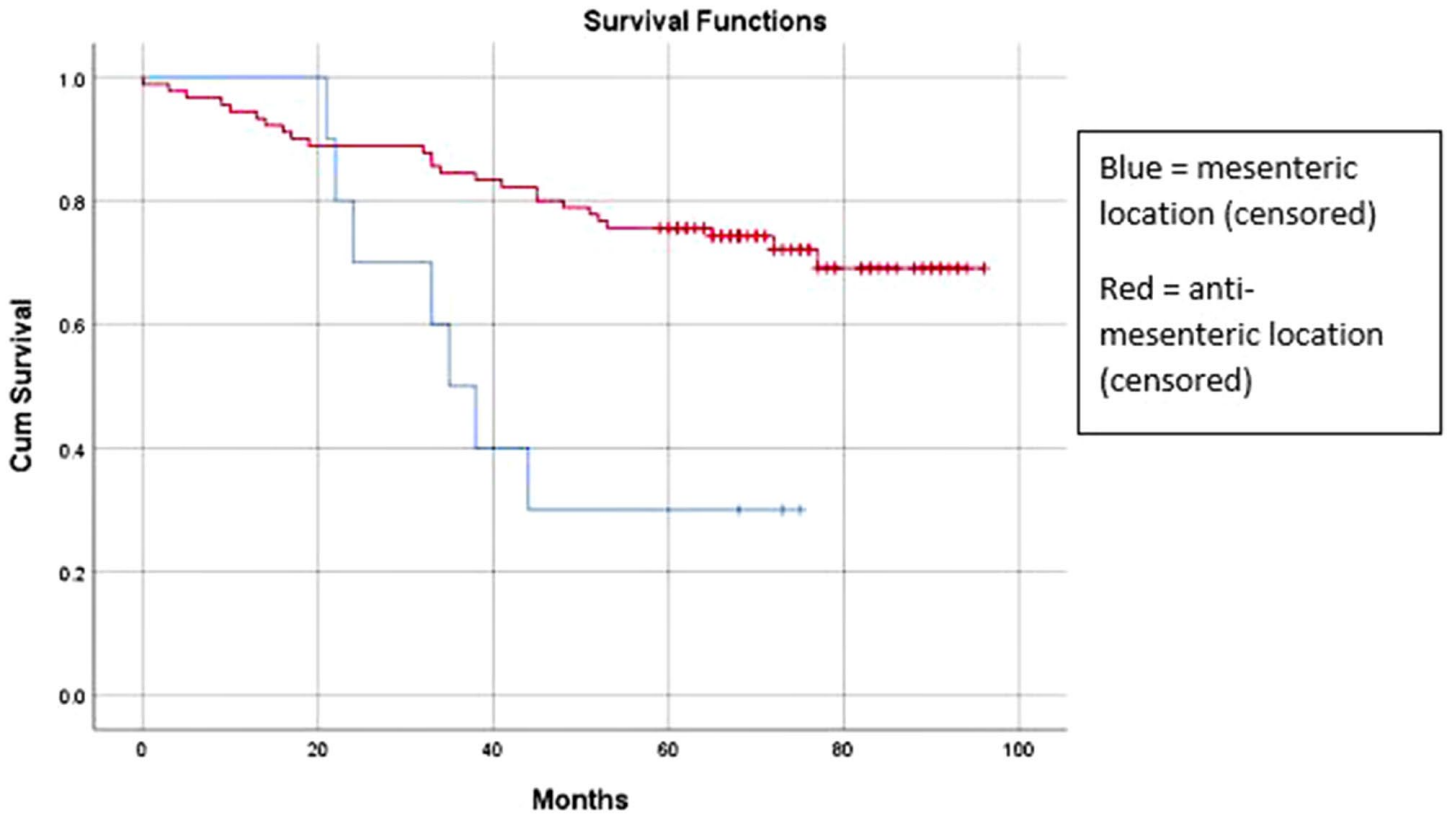
Kaplan–Meier survival graph.

The mean censored survival for the mesenteric group was 44.2 months, in comparison to 77.8 months in the anti-mesenteric group (*P* = 0.002), as shown in Fig. 3. The mean disease-free survival for the mesenteric group was 41.6 months, compared to 60.3 months (*P* = 0.021).

## Conclusion

This study demonstrates an all-cause mortality difference, with a worse prognosis for those with cancer on the mesenteric aspect of the right colon. We could not demonstrate a significant difference in lymph node involvement or T stage between the two groups, unlike the two previously described studies. Although not statistically significant, the antimesenteric group was more represented in the higher T and N stages and perhaps could be masking a greater survival difference between the two groups. Age and sex were equally matched and therefore not deemed to be an influencing factor.

## Discussion

Having performed a literature search on PubMed, Google Scholar & The Cochrane Library there is no study to date comparing survival of mesenteric versus anti-mesenteric in right sided colon cancer. A recent study published in 2017 demonstrated an increase in lymph node involvement in colorectal cancer when the tumour was on the mesenteric aspect (this included both left and right cancer)^16^. There are two other small scale, contemporary, studies from Italy also hinting that mesenteric cancers have a higher lymph node rate when compared to anti-mesenteric cancers, but importantly they were unable to demonstrate a survival difference^17,18^. It is also worth noting that the concept is so novel that the mesenteric location is not a required dataset for pathologists to be reporting on^19^.

This pilot study is limited in a number of ways: the sample size is small, it excludes left sided cancer and known oncological prognostic factors were not recorded or included in a multivariate analysis (lymphovascular invasion, cancer genetics, chemotherapy use etc.), all of which will have an influence on mortality.

Moreover, at present, there are no standardised guidelines on pathological reporting of tumour location, other than in rectal cancer. This meant that by using historical pathology reports, we were relying on subjective descriptive terms and applying it to our definition (Fig. 1). This therefore opens the possibility that the results are heterogenous. The author is currently evaluating the accuracy of pre-operative radiology on contemporary histology reports using our mesenteric definition. Should this show an acceptable level of precision, it will allow a larger retrospective study on historical data, this time incorporating more data points.

We elected to exclude metastatic disease from this pilot study as we hypothesised that by keeping the focus on early-stage disease, the survival difference in our small sample size was more likely to elicit a greater difference than in those whom have already demonstrated systemic spread. It is, however, possible that anti-mesenteric cancers are more ‘aggressive’ than mesenteric ones, and are higher represented in this excluded metastatic sub-category of patients.

This study did not find a statistically significant difference in nodal stage between the anti-mesenteric and mesenteric groups—which is divergent to the Italian and Polish studies^16,17^. We cannot elucidate why this is, and if the hypothesis is correct, we would expect a higher lymph node involvement rate in the mesenteric group. With ten patients, this group is certainly underpowered which could explain why our data differs to that of the aforementioned studies.

Despite the shortcoming of this study, the results are in keeping with a growing body of evidence that mesenteric tumours act differently to anti-mesenteric ones. This is an encouraging sign for a possible prognostication factor that we have been neglecting. Further study is needed to clarify this potential relationship. Should future studies show similar survival results, potential thought in the onco-surgical community may have to be given to randomised control trials incorporating more aggressive treatment paradigms for mesenteric cancers—for example chemotherapy to all mesenteric cancer, even in low stage disease.

## Data Availability

Any data, methods or study materials are available to other researchers on request.
